# Body Composition and Metabolic Improvement in Patients Followed Up by a Multidisciplinary Team for Obesity in China

**DOI:** 10.1155/2021/8862217

**Published:** 2021-07-29

**Authors:** Difei Lu, Zhenfang Yuan, Lihua Yang, Yong Jiang, Min Li, Yuanzheng Wang, Lulu Jing, Rongli Wang, Junqing Zhang, Xiaohui Guo

**Affiliations:** ^1^Department of Endocrinology, Peking University First Hospital, China; ^2^Department of Clinical Nutrition, Peking University First Hospital, China; ^3^Department of General Surgery, Peking University First Hospital, China; ^4^Department of Rehabilitation, Peking University First Hospital, China; ^5^Department of Integrative Traditional Chinese Medicine and Western Medicine, Peking University First Hospital, China

## Abstract

**Background:**

This study evaluated the effectiveness of the multidisciplinary team (including a specialist, a dietitian, a physical exercise trainer, a surgeon for bariatric surgery, an acupuncturist, and several health educators) for obesity management and the body composition change and improvements in metabolic biomarkers during a 2-year follow-up.

**Materials and Methods:**

A total of 119 patients participated in the multidisciplinary team for obesity. Patients were followed up at 3 months, 6 months, 1 year, 18 months, and 2 years after their first visit. Individuals were divided into the high-protein diet (HPD) and standard-protein diet (SPD) group according to their results on a diet questionnaire that they filled out during follow-up.

**Results:**

After 1.2 years, the mean body weight of the participants dropped from 89.7 kg to 80.9 kg (*p* < 0.001). The body adiposity index was reduced from 33.9 to 32.0 (*p* < 0.001), while the fat-free mass index from 17.0 to 15.2 (*p* = 0.043). Fasting glucose and HbA1c were also lower after treatment (*p* = 0.002 and 0.038 for FPG and HbA1c, respectively). Fasting insulin and HOMA-IR were reduced (*p* = 0.002 and <0.001 for fasting insulin and HOMA-IR, respectively). HDL-c increased along with weight loss (1.06 mmol/L vs. 1.19 mmol/L, *p* < 0.001), and transaminase levels significantly dropped (*p* = 0.001 and 0.021 for ALT and AST, respectively). During treatment, mean protein intake was 29.9% in the HPD group and 19.5% in the SPD group (*p* < 0.001). Weight loss, reduction of visceral fat area, maintenance of lean body mass, body adiposity index, and fat-free mass index showed no statistical significance between the HPD and SPD groups, as well as glucose metabolic variables.

**Conclusions:**

A multidisciplinary team for obesity management could significantly reduce body weight and improve metabolic indicators, including HDL-c, transaminase, and insulin resistance. A high-protein diet does not produce better weight control or body composition compared with a standard calorie-restricted diet.

## 1. Introduction

The prevalence of obesity has been gradually increasing over the past 30 years. According to the most recent global estimates, over 500 million individuals are obese, which is creating a heavy health burden on patients and the social economy [1]. The most common complications of obesity include type 2 diabetes, hypertension, and hyperlipidemia. Regular physical activity, healthy diets, weight-loss medicines, and bariatric surgery are common treatments for obesity [2]. Moreover, a recent study proved that acupuncture might be effective against obesity [3]. Besides lifestyle changes, behavior intervention, such as patient education, motivating to a straightforward goal setting, and self-monitoring, is also of great importance [4]. Therefore, a multidisciplinary team that comprises physicians, dietitians, health educators, and physical activity trainers is recommended to execute lifestyle intervention for obesity patients in a more efficient way [5]. A consecutive follow-up by a multidisciplinary team can modulate an individualized lifestyle intervention, based on clinical evidence [6–8].

Some randomized controlled trials showed that a protein-rich hypocaloric diet is effective in reducing weight [9]. Other studies suggest that high-protein intake could increase satiety and reduce calorie intake. The proportion of macronutrients that a patient should take in order to reduce weight depends on individual goals, activity level, age, health, genetics, and much more.

The aim of this study was to evaluate the effectiveness of the multidisciplinary team for obesity management and investigate the body composition change and the improvements of metabolic biomarkers during a 2-year follow-up between a high-protein diet and a standard low-calorie diet. Our multidisciplinary outpatient clinic for obesity patients, which was launched on June 1, 2015, in Peking University First Hospital (PKUFH), includes an endocrinology specialist, a dietitian, a physical exercise trainer, a surgeon for bariatric surgery, an acupuncturist, and several health educators.

## 2. Materials and Methods

### 2.1. Study Design

Male and female individuals aged 18-75 years old with BMI > 24 kg/m^2^ defined as overweight and those >28 kg/m^2^ defined as obese were enrolled in the MDT clinic. All participants had given written informed consent at the first visit. Ethical approval was given from the Ethics Committee of Peking University First Hospital.

Diseases of secondary obesity, including Cushing's disease and hypothyroidism as exclusion criteria, were ruled out at the first visit. A series of educational programs were provided to the patients at the first visit to explain the goal of the study and the purpose of the treatment. During their first visit, participants' body height (BH), body weight (BW), waist circumference (WC), and hip circumference (HC) were measured and recorded by health educators. Demographic data, including age, gender, smoking status, daily food intake, and past medical history (type 2 diabetes, hypertension, hyperlipidemia, hyperuricemia, and nonalcoholic fatty liver disease (NAFLD)), were analyzed through a questionnaire.

After the anthropometric evaluations, a fasting venous blood sample was withdrawn to obtain baseline data for a full-scale evaluation of weight-related comorbidities, including liver function, lipid profile, HbA1c, fasting plasma glucose, plasma uric acid, and fasting plasma insulin.

Body fat percentage (BF%), lean body mass (kg), muscle mass of lower extremities (kg), and visceral fat area (cm^2^) along with other body composition variables were determined using bioelectrical impedance analysis (InBody® 720 Medical Body Composition Analyzer, Biospace Co., Ltd., Korea). Body adiposity index (BAI) was calculated from height and hip circumference (HC) as follows: BAI = (HC (cm)/height (m)^1.5^) − 18 [10]. The fat-free mass index (FFMI) was calculated in accordance with dividing the lean mass (kg) by height squared (m) [11]. The homeostasis model assessment for insulin resistance (HOMA-IR) was calculated from fasting plasma glucose (FPG, mmol/L) and fasting insulin (mU/mL) using the following equation: HOMA − IR = fasting insulin × FPG/22.5 [12]. HOMA-*β* for *β*-cell function was determined with the equation HOMA − *β* = (20 × fasting insulin)/(FPG − 3.5) [12].

### 2.2. Diets and Intervention

At each visit, patients met with the whole multidisciplinary team, which consisted of a physician, a dietitian, an exercise trainer, a surgeon for bariatric surgery, an acupuncturist, and several health educators. An individualized meal plan and an exercise guide were provided at the initial visit, and pharmacotherapy or acupuncture procedure was recommended. If no significant weight reduction was observed three to six months after standard management, bariatric surgery was recommended.

Regular follow-up was scheduled at 3 months (V2), 6 months (V3), 1 year (V4), 18 months (V5), and 2 years (V6) after the initial visit (V1). Body weight and recent food intake were recorded at each visit by the nursing staff. Patients were enrolled from June 2015 to June 2017, and they were regularly followed up by the multidisciplinary team. Individualized management was modulated at every visit for each patient. A high-protein diet was defined as daily protein intake above 20% of total calorie intake, in the meantime meeting the criteria of exceeding1.5 g/kg body weight/day. Patients were allocated to a standard protein diet (SPD) group and a high-protein diet (HPD) group based on their food intake proportion during follow-ups.

### 2.3. Statistical Analysis

SPSS version 18.0 (IBM, USA) was used for analyses. Continuous variables were described as mean ± standard deviation (SD) and categorical variables as a percentage. A paired *t*-test was used to compare within-group mean differences between visits. A chi-square test for independence was used to compare the differences between categorical variables. Student's *t*-test was used for means of metabolic indicators between HPD and SPD groups.

## 3. Results

### 3.1. Baseline Characteristics

A total of 119 patients received integrated care and follow-up until June 2017. Among them, 32 (26.9%) patients were prescribed metformin, 16 (13.4%) patients received acupuncture therapy, and 3 (2.7%) patients received glucagon-like peptide 1 (GLP-1), an agonist. There was no significant difference in baseline characteristics and anthropometric data between high-protein diet (HPD) and standard-protein diet (SPD) groups at their first visit (Table 1). The majority of the patients were female (72.3%) and young (aged from 26 to 52 years old). The mean body weight at the initial visit was 89.7 ± 20.1 kg, and the mean body mass index (BMI) was 32.7 kg/m^2^. Their mean follow-up duration was 1.2 years. There were high comorbidity rates of metabolic diseases. Among the patients, 38.8% suffered hyperlipidemia, 39.5% had type 2 diabetes, 37.4% had hypertension, 60.4% had nonalcoholic fatty liver disease (NAFLD), 21.4% had hyperuricemia, and 38.7% had metabolic syndrome.

### 3.2. Improvements in Body Weight and Metabolic Indicators

After a 1.2-year mean duration of multidisciplinary treatment in PKUFH, the mean body weight of the participants dropped from 89.7 kg to 80.9 kg (*p* < 0.001), accompanied with the loss of lean body mass of 1.2 kg (51.7 kg vs. 50.5 kg, *p* < 0.001) and lower extremity muscle mass of 0.4 kg (16.1 kg vs. 15.7 kg, *p* < 0.001). Body adiposity index was significantly reduced from 33.9 to 32.0 (*p* < 0.001), while fat-free mass index from 17.0 to 15.2 (*p* = 0.043).

Regarding glucose metabolism, the distinctive reduction of FPG and HbA1c indicated improved glucose control after treatment (*p* = 0.002 and 0.038 for FPG and HbA1c, respectively, Table 2). Also, predominance change was discovered in insulin resistance based on the decline of fasting insulin level and HOMA-IR (*p* = 0.002 and <0.001 for fasting insulin and HOMA-IR, respectively, Table 2). Pancreatic *β*-cell function did not display a significant change (*p* = 0.134 for HOMA-*β*).

Concerning lipid metabolism, HDL-c remarkably increased along with weight loss (1.06 mmol/L vs. 1.19 mmol/L, *p* < 0.001), but triglyceride, TCHO, and LDL-c remained unchanged. There was a high comorbidity rate of NAFLD in the participants, and the transaminase levels were significantly reduced after weight management (*p* = 0.001 and 0.021 for ALT and AST, respectively, Table 2).

### 3.3. Weight Loss in HPD and SPD Groups

During the diet, the mean calorie intake was 1389 kcal/d in the HPD group with 29.9% protein proportion and 1393 kcal/d in the SPD group with a 19.5% protein proportion (*p* < 0.001 for protein intake percentage, Table 3). After the mean treatment duration of 1.2 years in obesity MDT in PKUFH, weight loss of 9 kg was observed in both groups. According to the guidelines on obesity, weight loss of 5% to 10% is the first goal of lifestyle intervention treatment [1]. Defining body weight reduction over 5% of the baseline value as successful weight loss, the success rate was 77.8% in the HPD group and 68.5% in the SPD group (*p* = 0.351). Considering the reduction of body weight over 10% as successful weight loss, the rate of success was 48.1% in the HPD group and 34.8% in the SPD group (*p* = 0.208). The reduction of visceral fat area, maintenance of lean body mass, body adiposity index, and fat-free mass index showed no significance between the HPD and SPD groups, as well as glucose metabolic variables including HOMA-IR and HOMA-*β* (Table 3).

## 4. Discussion

This study provides insight into the effectiveness of applying a multidisciplinary team for weight-loss intervention in the real world. In order to explore the effectiveness of a group-based weight-loss clinic, body weight was assessed during the follow-up. Overall, our results revealed that body weight significantly decreased across the meantime span of 1.2 years of MDT treatment. It also demonstrated that a multidisciplinary weight-loss intervention could be more efficient in improving weight control and metabolic variables than traditional methods.

The obesity mechanism is complex. Besides genetic and environmental factors, the involvement of the opioid and endorphinergic systems has a critical role in feeding behavior, which brings new insight for future obesity medication [13]. In addition, cytokines, including adiponectin, contribute to the pathogenesis of insulin resistance in individuals with normal glucose tolerance and the development of obesity [14].

Over recent years, the lack of knowledge and awareness of obesity has become the main barrier to weight control, especially in China. Other barriers include a lack of motivation and previous negative experience about lifestyle intervention [15]. Our multidisciplinary team for obesity in Peking University First Hospital provides regular obesity education programs for obese patients, where peer support activities are regularly held since the first visit. Previous randomized controlled trials indicated that behavioral intervention could improve weight loss [16, 17]. Among the patients in our study, 68.1% received only education programs, a calorie-reduced meal plan, and an exercise training guide. A significant weight reduction was achieved without pharmacotherapy or bariatric surgery. In Gong et al.'s diabetes prevention study in China, body weight dropped over the 23-year follow-up with lifestyle intervention [18]. The participants in our study achieved the mean weight loss of 8.8 kg, including 1.2 kg loss of lean body mass, and 0.4 kg loss of lower extremity muscle mass that was maintained through the whole follow-up duration. Medication for weight reduction and glycemic control, including metformin, orlistat, and liraglutide, was prescribed to 31.9% of the patients in our study. A prospective study proved that metformin could reduce weight and cardiovascular risk by reducing endothelial dysfunction [19].

Body adiposity index (BAI), which is calculated from the hip circumference and height, is considered as a reliable scale that can directly estimate the percentage of adiposity, with higher sensitivity and specificity than traditional anthropometric parameters [20]. A previous study suggested that a lean body mass, especially the muscle mass of lower extremities, was positively associated with a longer life span [21]. During weight loss treatment, both adipose tissue and lean body mass were reduced over time. Our aim was to lower adipose mass and simultaneously keep lean body mass through a high-protein diet and resistance exercise training. Our results revealed a subtle loss of lean body mass, fat-free mass index (FFMI), and lower extremity muscle mass accompanied by a significant reduction of BAI and body weight.

At the initial visit, anthropometric data and metabolic parameters of the patients were collected. Among 119 patients (26 to 52 years old), 38.8% of patients suffered from hyperlipidemia, 39.5% from type 2 diabetes, 37.4% from hypertension, and 60.4% from NAFLD. Previous data suggested that obesity and high-fat meal could result in the increase of tumor necrosis factor *α* (TNF-*α*) and endothelial dysfunction [22], which may lead to the deterioration of metabolic indicators. Also, patients with diabetes have a higher risk of atherosclerosis progression, which might be related to the ubiquitin-proteasome system [23]. In our study, metabolic indicators, including HDL-c, transaminase, FPG, fasting insulin, and HOMA-IR, were improved along with weight loss. The reversing effect of insulin resistance in the participants indicated the necessity of regular screening for diabetes in obese patients with chronic status of insulin resistance and the urgency of weight loss.

A previous study has proven that common mechanisms, including endoplasmic reticulum stress, contribute to NAFLD and obesity [24]. In the present study, the occurrence of NAFLD in our study population reached 60.4%, while the prevalence of NAFLD in the world population was around 16% [25]. Along with the elevating body weight and BMI, the prevalence of nonalcoholic steatohepatitis (NASH), which is characterized by increased transaminase, could be further lifted. Once the transaminase is above the upper limit, a possibility of cirrhosis reaches 5-8% in 5 years of follow-up [26]. For both NASH and NAFLD, aside from lipid-lowering agents, one of the key management approaches was weight reduction [27]. Corroborating our findings, transaminase could be reduced after moderate weight loss and was beneficial for the management of NASH and NAFLD, as well as the prevention of cirrhosis.

Subgroup analysis of the high-protein diet and standard calorie-restricted diet was performed in our study to investigate the weight loss efficiency and metabolic alternation of the two diets. Previous studies, including some small scale randomized controlled studies (RCT), reported controversial conclusions about HPD. High-protein content in a diet resulted in more satiety, shrinking energy intake, and greater dietary thermogenesis. In an RCT in patients with metabolic syndrome, no significant differences in weight loss and biomarkers of metabolic syndrome were examined. Still, the participants with a higher adherence rate in the HPD group lost significantly more weight than those in the SPD group [28]. Patients in our study were recommended high-protein meal plans if they had a normal renal function at the first visit and were assigned to the HPD or SPD group in accordance with the diet questionnaire at each visit when daily protein intake was above 20% of the total calorie intake of the HPD group. Therefore, the number of participants in the HPD group was relatively small and weakened the statistical significance of weight reduction and glucose metabolic variables when comparing the two groups. The rate of weight loss over 5% was 77.8% in the HPD group and 68.5% in the SPD group (*p* = 0.351), and the rate of losing weight over 10% was 48.1% in the HPD group and 34.8% in the SPD group (*p* = 0.208). There was a tendency, but there was no statistical difference in weight loss efficiency in the HPD group; hence, no predominance of metabolic indicators in HPD was discovered in our study. A larger population and further study of the obesity MDT in PKUFH could elaborate more on the possible advantage of HPD.

This study has a few limitations. First, the data and results were collected from real-world cohort data, and it was impossible to avoid confounding factors that might affect the reliability of the conclusion. During our study, patients received a multidisciplinary treatment. For example, it was common that a patient underwent lifestyle intervention and acupuncture for a couple of months and was put on metformin for a year and then finally decided to have a bariatric surgery. Herein, the proportion of each kind of treatment was recorded in our study, and it was difficult to perform statistical analysis for the effectiveness of different ways for weight reduction. The follow-up duration and sample size were relatively small in our study. Continuous follow-up and patient enrollment, as well as a longer duration of follow-up and enlarged sample size, may further corroborate our results.

## 5. Conclusions

A multidisciplinary team for obesity management could significantly reduce body weight and improve metabolic indicators, including HDL-c, transaminase, and insulin resistance. A high-protein diet does not produce better weight control or body composition compared with a standard calorie-restricted diet.

## Figures and Tables

**Table 1:** Comparison of the demographic, anthropometric, and biochemical variables between SPD and HPD groups.

	HPD group (*n* = 27)	SPD group (*n* = 92)	*p* value^∗^
Age (years)	36.1 ± 9.7	40.0 ± 12.1	0.123
Sex category (male/female)	6/21	29/63	0.351
Follow-up duration (years)	1.1 ± 0.7	1.2 ± 0.6	0.339
Height (cm)	164.7 ± 8.5	164.9 ± 8.9	0.924
Weight (kg)	89.4 ± 19.9	89.8 ± 20.5	0.930
BMI (kg/m^2^)	32.7 ± 5.5	32.7 ± 5.3	0.951
Waist circumference (cm)	99.6 ± 16.1	100.5 ± 12.4	0.765
Hip circumference (cm)	109.9 ± 11.7	109.9 ± 10.8	0.989
Body fat (%)	41.0 ± 6.9	41.1 ± 5.5	0.889
Systolic blood pressure (mmHg)	126.8 ± 15.5	127.9 ± 16.3	0.766
Diastolic blood pressure (mmHg)	81.1 ± 6.7	82.4 ± 10.3	0.530
History of T2DM (*n*, %)	10, 37.0%	37, 40.2%	0.766
Fasting plasma glucose (mmol/L)	6.4 ± 2.4	6.3 ± 2.0	0.832
HbA1c (%)	6.8 ± 1.8	6.8 ± 1.7	0.974
Fasting insulin (*μ*IU/mL)	16.6 ± 9.5	19.6 ± 10.5	0.251
Fasting C-peptide (ng/mL)	2.9 ± 1.0	3.3 ± 1.2	0.138
HOMA-*β*	209.4 ± 59.1	198.0 ± 22.0	0.825
HOMA-IR	4.7 ± 3.2	5.1 ± 3.0	0.679
ALT (U/L)	41.9 ± 33.6	39.2 ± 33.3	0.744
AST (U/L)	26.7 ± 15.7	26.2 ± 16.2	0.891
Uric acid (*μ*mol/L)	364.1 ± 97.6	374.9 ± 95.7	0.639
Baseline calorie intake (kcal/d)	1681.9 ± 329.5	2141.2 ± 651.6	0.037
Protein intake proportion (%)	16.4 ± 3.2	17.2 ± 2.6	0.875
Fat intake proportion (%)	31.6 ± 7.0	33.3 ± 7.5	0.532
Glucose intake proportion (%)	52.2 ± 8.2	52.4 ± 7.2	0.955
Visceral fat area (cm^2^)	173.8 ± 46.0	174.3 ± 40.2	0.960
Lean body mass (kg)	52.3 ± 11.3	52.3 ± 12.0	0.989
Muscle mass of lower extremities (kg)	16.3 ± 3.4	16.2 ± 4.0	0.851
Body adiposity index	34.1 ± 5.1	34.0 ± 4.8	0.901
Fat-free mass index	16.7 ± 6.4	17.5 ± 5.6	0.538

T2DM: type 2 diabetes mellitus; ALT: glutamic-pyruvic transaminase; AST: glutamic-oxaloacetic transaminase. ^∗^*χ^2^* test for categorical variables and Student's *t*-test for continuous variables.

**Table 2:** Variation of anthropometric and metabolic indicators after treatment of obesity MDT.

	*n*	Baseline	After follow-up	*p* value^∗^
Weight (kg)	119	89.7 ± 20.0	80.9 ± 17.1	<0.001
Lean body mass (kg)	119	51.7 ± 10.9	50.5 ± 10.0	<0.001
Hip circumference (cm)	119	109.9 ± 10.8	105.8 ± 9.5	<0.001
Muscle mass of lower extremities (kg)	119	16.1 ± 3.6	15.7 ± 3.4	<0.001
Body adiposity index	119	33.9 ± 4.9	32.0 ± 4.3	<0.001
Fat-free mass index	119	17.0 ± 6.2	15.2 ± 7.2	0.043
Fasting plasma glucose (mmol/L)	119	6.0 ± 1.5	5.5 ± 0.9	0.002
Fasting insulin (*μ*IU/mL)	119	19.0 ± 10.8	13.8 ± 6.6	0.002
HOMA-IR	119	4.8 ± 2.6	3.2 ± 1.5	<0.001
HOMA-*β*	119	256.7 ± 39.1	202.9 ± 21.4	0.134
HbA1c (%)	34	6.89 ± 0.26	6.33 ± 0.93	0.038
ALT (U/L)	54	36.0 ± 3.3	24.7 ± 2.2	0.001
AST (U/L)	53	23.8 ± 1.6	20.1 ± 1.0	0.021
Uric acid (*μ*mol/L)	53	359.0 ± 91.9	351.6 ± 74.6	0.480
Triglyceride (mmol/L)	54	2.15 ± 0.23	2.06 ± 0.37	0.745
TCHO (mmol/L)	54	4.70 ± 0.14	4.86 ± 0.17	0.320
HDL-c (mmol/L)	54	1.06 ± 0.26	1.19 ± 0.30	<0.001
LDL-c (mmol/L)	54	2.86 ± 0.96	2.85 ± 0.88	0.963

ALT: glutamic-pyruvic transaminase; AST: glutamic-oxaloacetic transaminase; TCHO: total cholesterol; HDL-c: high-density lipoprotein cholesterol; LDL-c: low-density lipoprotein cholesterol. ^∗^*χ^2^* test for categorical variables and Student's *t*-test for continuous variables.

**Table 3:** Comparison of diet, anthropometric and biochemical variables between SPD and HPD group after treatment of obesity MDT.

	HPD group (*n* = 27)	SPD group (*n* = 92)	*p* value^∗^
Calorie intake during diet (kcal/d)	1389.0 ± 440.9	1393.0 ± 431.5	0.966
Protein intake proportion during diet (%)	29.9 ± 6.3	19.5 ± 3.6	<0.001
Weight after follow-up (kg)	80.4 ± 17.7	80.8 ± 17.2	0.917
Weight reduction (kg)	9.0 ± 1.5	9.0 ± 1.1	0.992
Percentage of weight loss (%)	9.8 ± 1.5	9.3 ± 0.9	0.780
Weight loss > 5% (*n*, %)	21, 77.8%	63, 68.5%	0.351
Weight loss > 10% (*n*, %)	13, 48.1%	32, 34.8%	0.208
Hip circumference (cm)	105.1 ± 10.3	106.0 ± 9.4	0.684
Visceral fat area (cm^2^)	131.8 ± 54.7	134.7 ± 43.6	0.797
Lean body mass (kg)	49.5 ± 9.8	50.2 ± 9.9	0.744
Muscle mass of lower extremities (kg)	15.3 ± 3.4	15.6 ± 3.4	0.737
Body adiposity index	31.7 ± 4.0	32.2 ± 4.3	0.652
Fat-free mass index	14.9 ± 7.4	14.9 ± 7.4	0.965
Fasting plasma glucose (mmol/L)	5.5 ± 1.0	5.6 ± 0.9	0.726
Fasting insulin (uIU/mL)	12.8 ± 7.9	14.0 ± 6.2	0.615
HOMA-*β*	71.9 ± 25.5	54.0 ± 11.6	0.485
HOMA-IR	1.0 ± 0.3	1.0 ± 0.2	0.882

^∗^ *χ^2^* test for categorical variables and Student's *t*-test for continuous variables.

## Data Availability

Data is available at any time after contacting the corresponding author via email.
